# Birds, blooms, and evolving diversity

**DOI:** 10.1371/journal.pbio.3000020

**Published:** 2018-10-04

**Authors:** Lauren A. Richardson

**Affiliations:** Public Library of Science, San Francisco, California, United States of America

## Abstract

In this Open Highlight, Senior Editor Lauren Richardson features exciting new Open Access research into how species evolve their characteristic traits.

Every species can be defined by a set of traits. These traits can be attributes that help it to attract mates (such as bird songs) or aid in accessing a resource (like the long beak of the hummingbird) or any other element that describes their form or function. How these different traits develop and diversify over time is a long-standing question in the field of evolutionary biology. In this Open Highlight, I will feature research from the Open Access corpus that investigates and sheds light on how species have evolved their characteristic traits.

In their *PLOS Biology* article, Jonathan Drury, Hélène Morlon, and colleagues study how competition between species within the tanager family, the largest family of songbirds, has shaped their trait evolution [1] (Fig 1). While the role of competition has been studied in the context of resource use for spatially constrained island-based species, such as Darwin’s finches, here they study a large radiation across Central and South America and include social traits such as plumage signals and song. Using an impressive data set and a novel framework, they show that competition strongly impacted the diversification of resource-use traits like beak morphology but had a far weaker effect on social signaling traits like song tempo and frequency. They observed that these social signaling traits evolved more rapidly, which fits with previous theory that these traits evolve during the phase of speciation when the newly forming species is geographically isolated from other closely related species. When secondary contact occurs between this new species and its relatives, the new species is thus sufficiently distinct in its signals to avoid social competition.

**Fig 1:**
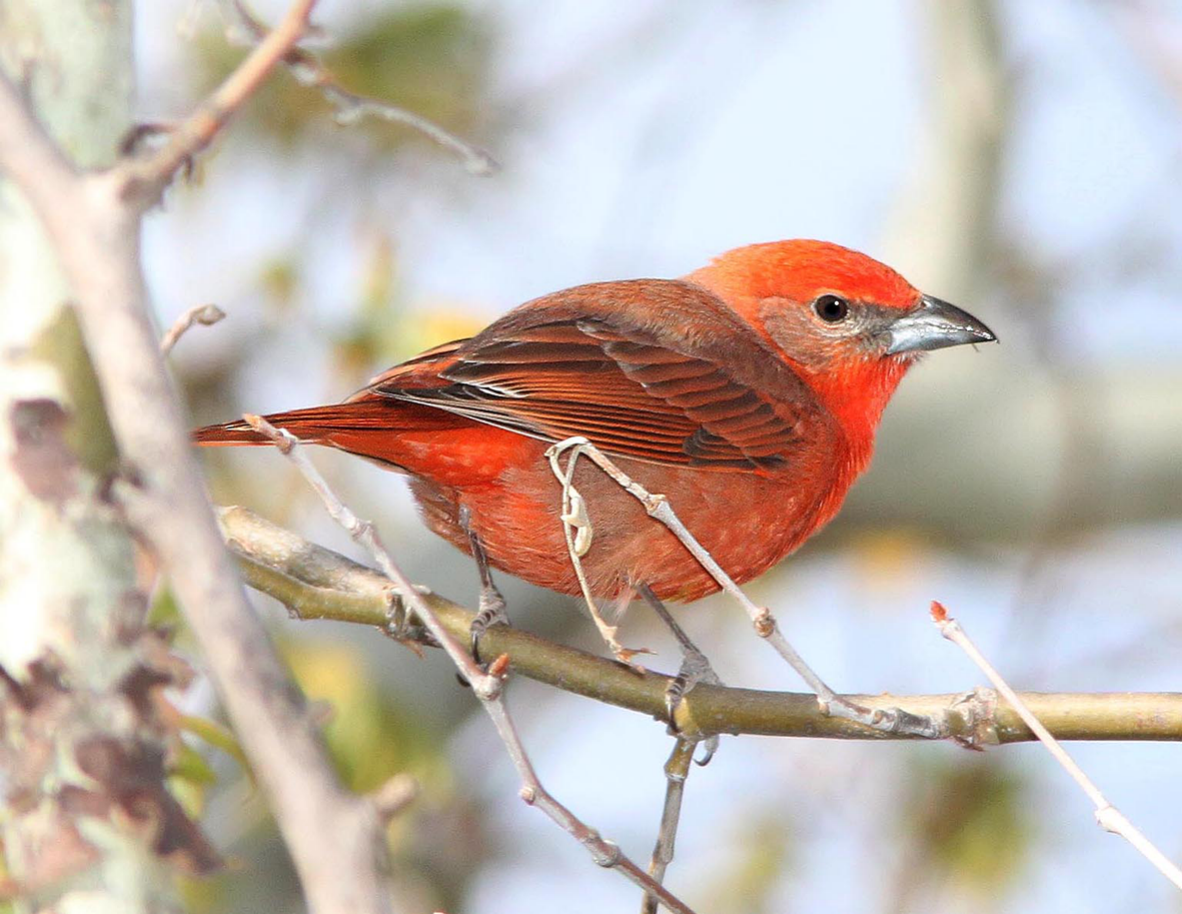
Hepatic tanager. Image credit: Flikr user Alan Schmierer.

Birds, with their showy mating routines and songs, are a popular subject for trait evolution studies. One of the foremost goals of research into trait radiation is identifying the genetic differences that lead to specific phenotypes. In this *Science Advances* article, the authors compare the genomes of nine species of birds (capuchino seedeaters) that live in overlapping regions of South American grassland [2]. Their genomes were highly similar, yet the authors found divergent areas, many of which contained genes involved in pigmentation, explaining their varied feather color.

Differences in environment can also drive trait evolution in both direct and indirect ways. In this *Ecology and Evolution* article, the authors tested whether the evolution of the ovenbird song was due to a direct influence of habitat characteristics or the indirect effect of body size and beak size, with their data supporting the latter scenario [3].

The evolution of some traits is complex and involves multiple steps. In this *PLOS Biology* article, the authors describe the evolution of cooperative breeding (in which nonfamily members share in brood care) in birds [4] (Fig 2). They find that birds first evolved family living with prolonged parent–offspring interactions, followed by a second step with the retained offspring helping at the nest. Thus, this family living scenario acted as a stepping stone to cooperative breeding, which they link to ecological resiliency.

**Fig 2:**
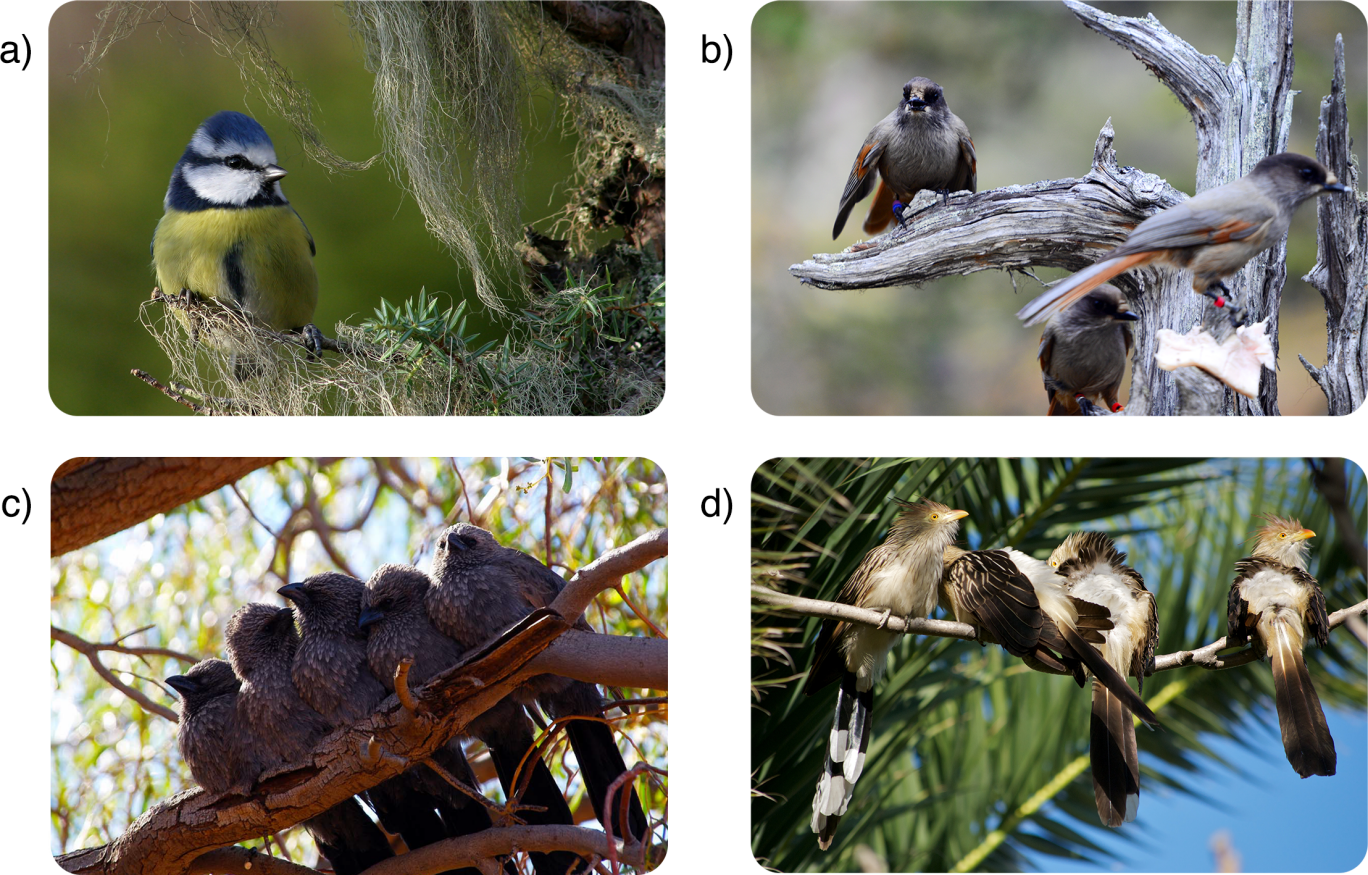
Species of birds with different social systems. (a) Blue tit *Parus caeruleus* young leave their parents after nutritional dependency ends and do not engage in cooperative breeding, (b) the Siberian jay *Perisoreus infaustus* is a family-living species without cooperative breeding, (c) apostlebird *Struthidea cinereal* offspring stay with their parents and help rear subsequent broods, and (d) the guira cuckoo *Guira* is an example of the rare case in which cooperative breeding involves nonkin individuals. *Image credit*: *doi*: *10*.*1371/journal*.*pbio*.*2000483*.

Whether related species co-occur in the same region and ecosystem depends on multiple factors, as described in this *PLOS ONE* article [5]. By probing data on hummingbird assemblages in the Northern Andes with a new model, they test how environment, dispersal, and competition impact the likelihood of co-occurrence.

Another group of organisms frequently used to study trait evolution is angiosperms or flowing plants. Angiosperms are an extremely successful and diverse group, and while flowers are extremely varied today, they all evolved from the same original flowering plant. But what did that first flower look like? In this article published in *Nature Communications*, researchers describe their work reconstructing the ancestral angiosperm flower and its subsequent diversification [6] (Fig 3).

**Fig 3:**
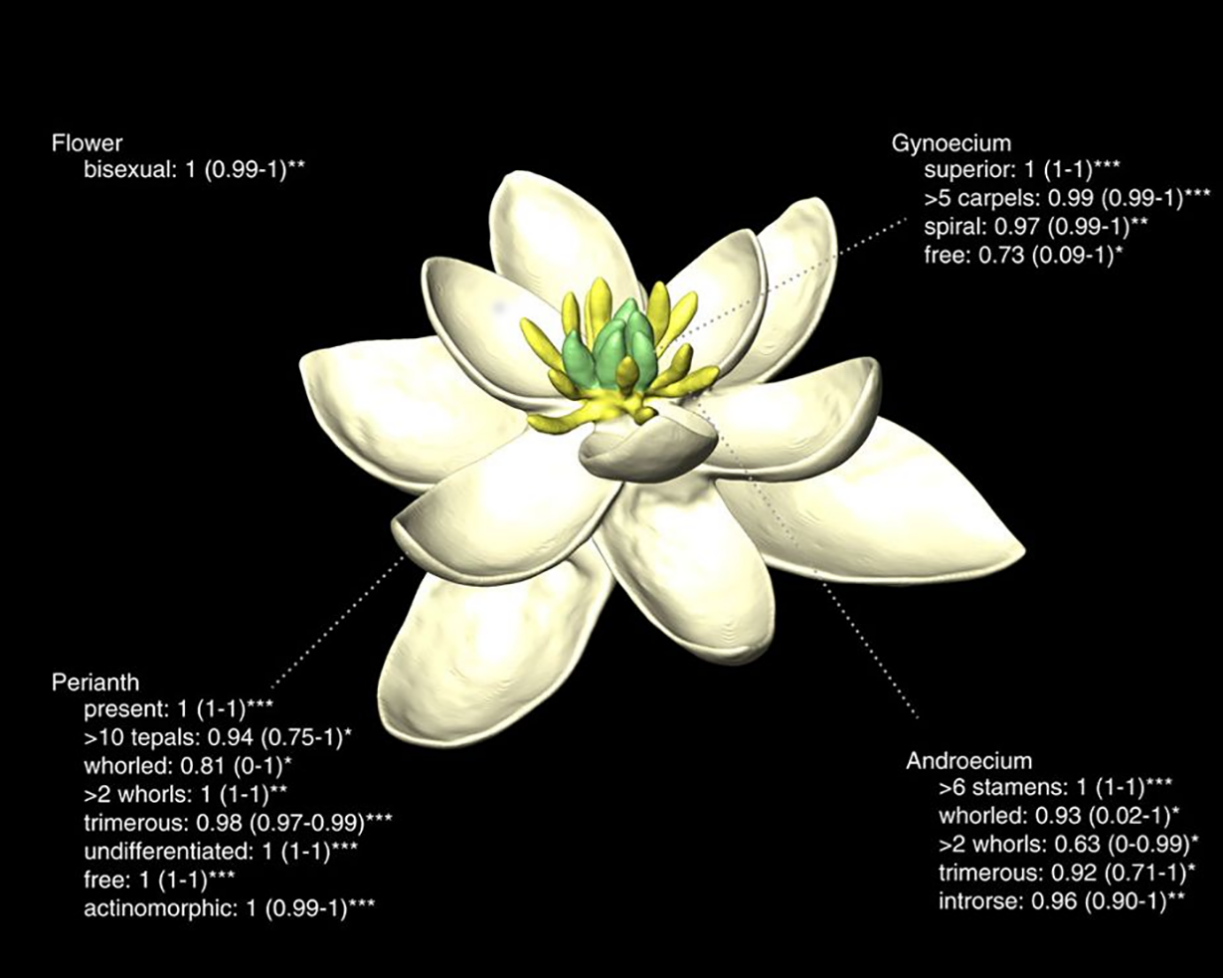
A reconstruction of the angiosperm ancestral flower. Image credit: doi: 10.1038/ncomms16047.

The evolution of traits always originates with genomic changes. In an article published in *PLOS Biology*, the authors describe how a systematic reduction in genome size may have led to the explosive success of angiosperms [7]. Smaller genomes mean smaller cells, allowing plants to pack more veins and stomata into a given leaf area, improving carbon dioxide (CO_2_) uptake and photosynthetic carbon gain, traits that allowed angiosperms to outcompete ferns and gymnosperms.

Within angiosperms, some groups are more diverse than others. Another *PLOS Biology* paper reveals how seed size correlates to rate of species formation, finding that faster rates of seed size change (which they use as a proxy for adaptability) are associated with faster speciation and thus more diversity within a group [8].

Before widespread genome sequencing, comparative analysis of gross anatomical traits was used to define the phylogenetic relationships of species. For angiosperms, this analysis was done on pollen morphology. In this *PLOS ONE* article, the authors present improved morphometric and morphospace methods to evaluate pollen change in the order of Myrtales [9]. This analysis allowed the authors to investigate patterns of convergent evolution and the effects of latitude.

It isn’t all just flowering plants and birds in the trait evolution field. While it is well established that snakes evolved from lizards, the initial ecological role of snakes is still unclear. The comparative analysis of the snake skulls described in this *Nature Communications* article suggests that snakes first evolved as fossorial or burrowing creatures, then diversified into surface and marine forms [10].

During speciation, lineages that have begun to diverge sometimes interact and begin to interbreed, which leads to introgression. In this *PLOS Genetics* paper, the authors identify introgression in species of Caribbean pupfish that influenced the evolution of jaw shape and size, eventually resulting in ability to utilize unique food resources [11].

Another *PLOS Genetics* article reconstructs evolutionary changes of gene expression phenotypes across multiple species of filamentous fungi to understand and identify recent shifts in gene function that impact the development of the sexual fruiting bodies [12].

For more detailed reading, please see the associated PLOS Collection [13].
